# Association between Glasgow Coma Scale in Early Carbon Monoxide Poisoning and Development of Delayed Neurological Sequelae: A Meta-Analysis

**DOI:** 10.3390/jpm12040635

**Published:** 2022-04-14

**Authors:** Myeong Namgung, Jaehoon Oh, Chiwon Ahn, Chan Woong Kim, Heekyung Lee, Hyunggoo Kang

**Affiliations:** 1 Department of Emergency Medicine, College of Medicine, Chung-Ang University, Seoul 06974, Korea; myeong15180@caumc.or.kr (M.N.); whenever@cau.ac.kr (C.W.K.); 2 Department of Emergency Medicine, College of Medicine, Hanyang University, Seoul 04763, Korea; ojjai@hanmail.net (J.O.); massdt@naver.com (H.L.); emer0905@gmail.com (H.K.)

**Keywords:** Glasgow Coma Scale, carbon monoxide intoxication, delayed neurological sequelae

## Abstract

A significant number of people experience delayed neurologic sequelae after acute carbon monoxide (CO) poisoning. The Glasgow Coma Scale (GCS) can be used to predict delayed neurologic sequelae occurrence efficiently and without any restrictions. Here, we investigated the association between a low GCS score observed in cases of early CO poisoning and delayed neurologic sequelae development through a meta-analysis. We systematically searched MEDLINE, EMBASE, and the Cochrane Library for studies on GCS as a predictor of delayed neurologic sequelae occurrence in patients with CO poisoning in June 2021. Two reviewers independently extracted study characteristics and pooled data. We also conducted subgroup analyses for the cutoff point for GCS. To assess the risk of bias of each included study, we used the quality in prognosis studies tool. We included 2328 patients from 10 studies. With regard to patients with acute CO poisoning, in the overall pooled odds ratio (OR) of delayed neurologic sequelae development, those with a low GCS score showed a significantly higher value and moderate heterogeneity (OR 2.98, 95% confidence interval (CI) 2.10–4.23, I^2^ = 33%). Additionally, in subgroup analyses according to the cutoff point of GCS, the development of delayed neurologic sequelae was still significantly higher in the GCS < 9 group (OR 2.80, 95% CI 1.91–4.12, I^2^ = 34%) than in the GCS < 10 or GCS < 11 groups (OR 4.24, 95% CI 1.55–11.56, I^2^ = 48%). An initial low GCS score in patients with early CO poisoning was associated with the occurrence of delayed neurologic sequelae. Additionally, GCS was quickly, easily, and accurately assessed. It is therefore possible to predict delayed neurologic sequelae and establish an active treatment strategy, such as hyperbaric oxygen therapy, to minimize neurological sequelae using GCS.

## 1. Introduction

Carbon monoxide (CO) poisoning is one of the major causes of both intentional and unintentional morbidity and mortality [1]. In the United States, approximately 50,000 people with CO poisoning visit the emergency department and over 400 of them die each year [2]. Additionally, according to the statistics of the Health Insurance Review & Assessment Service, more than 4000 cases of CO poisoning occur annually in South Korea [3].

The main pathophysiological mechanism of acute CO poisoning is hypoxia caused by compromised oxygen delivery. As inhaled CO, which has 200 times greater affinity for hemoglobin compared to oxygen, binds with hemoglobin to form carboxyhemoglobin, its oxygen-carrying capacity decreases [4]. The acute symptoms of CO poisoning range from nonspecific headache, dizziness, nausea, vomiting, and general malaise to altered mental status [5]. Patients with partial CO poisoning, after apparent recovery from acute symptoms, can experience neurological sequelae such as poor concentration, memory problems, personality changes, psychosis, and Parkinsonism [6]. These neurologic sequelae comprise delayed neurologic sequelae and are characterized by lucid intervals of 2–40 days [7]. According to Chambers et al., cognitive sequelae occurred in 34% of patients with CO poisoning [8], and Han et al. reported the incidence of delayed neurologic sequelae as 18.8% [9]. Although most patients with delayed neurologic sequelae improved over 1 year, approximately 15–25% of patients still exhibited neurologic deficits [10,11,12]. Since a significant number of patients with CO poisoning and delayed neurologic sequelae develop permanent impairments, it is important to predict delayed neurologic sequelae occurrence early for facilitating active and aggressive treatment. Previous studies investigating serum biomarkers have identified a relationship between troponin, S-100, creatinine kinase, or neuron-specific enolase and significant changes in delayed neurologic sequelae occurrence [9,13,14,15,16,17,18,19,20]. In addition, other studies have reported abnormal lesions related to delayed neurologic sequelae occurrence after performing early brain imaging [21,22,23]. However, because some medical institutions have restricted access to these biomarkers and brain imaging and because it takes time to confirm the results, a fast and commonly available test should be considered to predict the occurrence of delayed neurologic sequelae.

Several studies have suggested that initial altered mental status is a known risk factor for delayed neurologic sequelae development and have shown that its occurrence is higher in the group with a low Glasgow Coma Scale (GCS) score [11,24,25]. GCS is easier to use clinically than other resources in the emergency room because it can be quickly and simply assessed by clinicians. In the absence of a meta-analysis of the development of delayed neurologic sequelae in patients with acute CO poisoning and an early low GCS score, we performed such an analysis here.

## 2. Materials and Methods

### 2.1. Reporting Guidelines and Protocol Registration

We based this study on the Preferred Reporting Items for Systematic reviews and Meta-analyses and the Meta-analysis of Observational Studies in Epidemiology guidelines for reporting information from observational studies [26,27]. We prospectively registered the review protocol in the PROSPERO database (CRD42021241776).

### 2.2. Search Strategy

Two experienced reviewers (C.A. and J.O.) systematically searched three electronic databases (MEDLINE, Embase, and the Cochrane Library) for studies on GCS as a predictor of delayed neurologic sequelae occurrence in patients with CO poisoning through June 2021. We included medical subject headings (MeSH), Embase subject headings, and text words in our search strategy. We combined the MeSH terms and free terms related to “carbon monoxide”, “carbon monoxide poisoning”, and “delayed neurological sequelae”. We present the detailed search strategy in Supplementary Table S1.

### 2.3. Study Selection

Two reviewers (M.N. and C.A.) independently screened the titles, abstracts, and type of each of the identified articles, excluding irrelevant studies. First, we eliminated duplicate studies. If the title, author, and the publication year of a paper were the same, we judged it as a duplicate paper. We then excluded all articles meeting the following criteria: reviews, case reports, case series, editorials, letters, comments, conference abstracts, or meta-analyses; animal studies; irrelevant populations; and inappropriate controls. Among the papers published by the same title and the same author, we judged those published in a journal and those published as a conference abstract to be different. Where two reviewers disagreed regarding study selection, the third reviewer (J.O.) intervened, and differences were discussed until consensus was reached.

Ultimately, we included studies which assessed the initial GCS score and the development of delayed neurologic sequelae in acute CO poisoning. The clinical criterion by which we included studies to diagnose delayed neurologic sequelae was the development of neurological sequelae after a lucid interval. We excluded studies that: (1) included patients younger than 18 years, (2) included patients who failed to recover from a decreased mental status (that is, suffered permanent neurologic injury) or died, and (3) non-original articles. We subsequently reviewed the full text of potentially relevant articles that met the inclusion criteria.

### 2.4. Data Extraction

The two reviewers independently extracted the following information from the included studies: authors, year of publication, region of study, sample size, age, sex, initial GCS score, administration of hyperbaric oxygen therapy, and development of delayed neurologic sequelae. Discrepancies between reviewers were resolved by consensus. For the GCS score—the main outcome of our study—we extracted the odds ratio. When the included studies failed to present the odds ratio, we calculated it.

### 2.5. Risk of Bias Individual Studies

We assessed the methodological quality of the ten included studies using the quality in prognosis studies (QUIPS) tool [28]. Two reviewers (M.N. and J.O.) assessed the included ten studies independently; any unresolved disagreements between reviewers were resolved by discussion with the third author, with blinding to authorship and journal performed independently. We analyzed publication bias using the funnel plot and Egger’s test.

### 2.6. Statistical Analysis

The meta-analysis investigated the association between the initial low GCS score and the occurrence of delayed neurologic sequelae in patients with CO poisoning. For dichotomous variables, we calculated the pooled odds ratio with a 95% confidence interval (CI) using a random-effects model. We estimated the proportion of between-study inconsistency using the I^2^ statistic to assess heterogeneity, considering I^2^ values of 25%, 50%, and 75% as low, moderate, and high heterogeneity, respectively [29].

We conducted planned subgroup analyses based on sample size (>100 participants), the cutoff point of GCS (GCS < 9 points versus GCS < 10 or GCS < 11 points), and the quality of included studies. We performed a sensitivity analysis using sequential removal of individual studies and subsequent determination of an overall pooled approximation for the remaining studies.

We performed a meta-analysis and quality assessment of the included studies using Review Manager version 5.4 (Cochrane Collaboration 2012, Nordic Cochrane Centre, Copenhagen, Denmark) and R (version 4.0.0, The R Foundation for Statistical Computing, Vienna, Austria) software packages “meta” (version 4.11-0) and “metaphor” (version 2.1-0), respectively, considering a *p*-value of <0.05 to be statistically significant. We assessed publication bias using a funnel plot and Egger’s test.

## 3. Results

### 3.1. Study Selection

**Figure 1:**
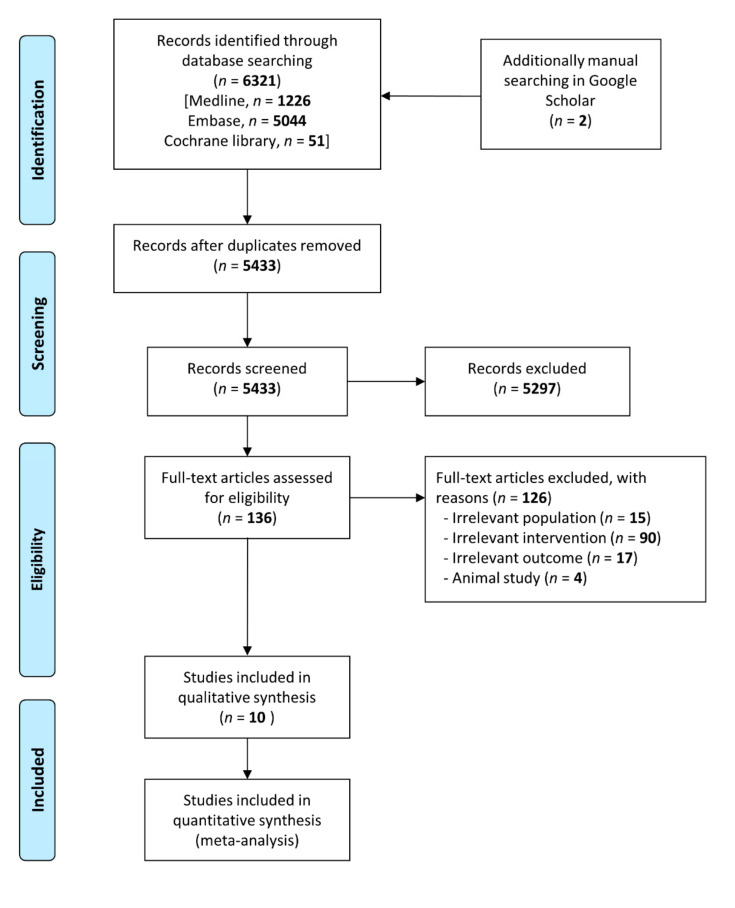
Flow diagram for identification of relevant studies.

Initially, we included 6323 studies and analyzed 136 papers by reviewing the title and the abstract. We excluded 126 studies because of irrelevant population (*n* = 15), irrelevant intervention (*n* = 90), irrelevant outcome (*n* = 17), and animal study (*n* = 4) (Supplementary Table S2). Finally, we included 10 eligible studies, including 2328 patients, in this analysis [1,6,9,17,19,25,30,31,32,33]. In Figure 1, we show the flow chart for identifying eligible studies.

### 3.2. Study Characteristics

We evaluated the initial GCS in 10 studies to predict delayed neurologic sequelae occurrence. The cutoff points of a low GCS score associated with delayed neurologic sequelae occurrence were 9, 10, and 11 in seven, two, and one studies, respectively. Eight studies were conducted in East Asia—including Korea, Japan, and Taiwan—and two studies were conducted in Italy and Egypt. Six studies were retrospective while four studies were prospective. All but one (multicenter) investigation were single-center investigations. The maximum follow-up period of the analyzed studies ranged from 6 weeks to 2 years. We summarize the characteristics of the included studies in Table 1.

### 3.3. Risk of Bias and Quality Assessment

We assessed the risk of bias of included studies with the QUIPS tool, evaluating all studies with low bias in three domains—including prognostic factor measurement, outcome measurement, and statistical analysis and reporting. In the domain of study attrition, because there were no studies which completely described follow-up loss or drop-out of participants during the study period, we rated all studies as unclear or high bias. We assessed studies with four or more low-bias domains in a total six domains as high-quality studies, with 6 of 10 studies reaching this benchmark [9,17,24,25,30,31]. We present details of our assessment of study quality in Supplementary Figure S1.

### 3.4. Main Analysis and Subgroup Analysis

**Figure 2:**
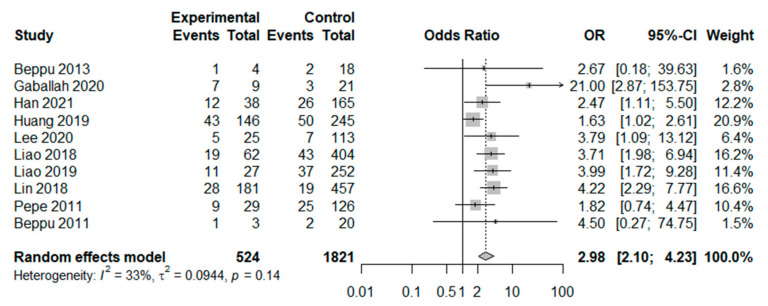
Forest plot for the effect of initial low Glasgow Coma Scale and occurrence of delayed neurological sequelae in carbon monoxide poisoning [1,6,9,17,19,25,30,31,32,33].

Among patients with acute CO poisoning, the overall pooled odds ratio of delayed neurologic sequelae development was significantly higher in the early low GCS score group than that in the high GCS group (odds ratio 2.98, 95% CI 2.10–4.23, I^2^ = 33%) (Figure 2). Additionally, we performed subgroup analyses according to sample size (≥100 versus <100), comparing the cutoff points of GCS (GCS < 9 points versus GCS < 10 or GCS < 11 points) and study quality (high versus low). Although we could not completely resolve the heterogeneity of included studies, in all subgroup analyses, the development of delayed neurologic sequelae was still significantly higher in the low GCS score group (Table 2).

### 3.5. Publication Bias

There was no significant asymmetry in the funnel plot, and we did not observe any publication bias in the included studies based on Egger’s regression test (*p* = 0.1883) (Supplementary Figure S2 and Table S3).

## 4. Discussion

In this systematic review and meta-analysis, we confirmed that an initial low GCS score is a significant predictor for the occurrence of delayed neurologic sequelae. In particular, we identified a cutoff GCS score for patients with CO poisoning and delayed neurologic sequelae. The incidence of delayed neurologic sequelae was significantly high not only in patients with a GCS score of <9 but also in those with a GCS score of <10 or <11.

Previous studies showed that several factors—including biomarkers and brain imaging—were potential predictors for the development of delayed neurologic sequelae. However, the wider use of these test tools is limited by situational and cost constraints and there is no one optimal test to predict delayed neurologic sequelae occurrence. For example, in some studies, the consideration of the presence of loss of consciousness during the acute CO poisoning phase and a longer duration of CO exposure as predictors of delayed neurologic sequelae occurrence [1,20,22,30,34] may be inaccurate because these factors are based on historical assumptions. According to other studies on biomarkers, an elevated serum level of creatinine kinase, neuron-specific enolase, S100B protein, and copeptin were early predictors of delayed neurologic sequelae occurrence [9,15,17,20,35]. Several studies also reported that the presence of acute brain lesions on diffusion-weighted imaging was significantly associated with delayed neurologic sequelae occurrence [21,22,23]. Although these blood and brain imaging tests are useful in predicting the occurrence of delayed neurologic sequelae in patients with CO poisoning, such tests, due to equipment or cost constraints, cannot be conducted universally. Moreover, the confirmation of these test results is time consuming. Considering these aspects, the use the initial GCS to predict delayed neurologic sequelae occurrence is extremely useful because it can be quickly, easily, and accurately assessed.

Altered GCS scores and delayed neurologic sequelae occurrence during CO poisoning is associated with CO-induced hypoxia. In the early stages of CO poisoning and reperfusion injury, hypoxia induces consciousness-related changes in the central nervous system [19,36]. It produces excessive reactive oxygen species, which cause cell damage due to their oxidative power, and induces neuron demethylation and lipid peroxidation [37,38]. Through this mechanism, pathological neuronal changes are observed in patients with delayed neurologic sequelae, especially in the cerebral white matter and globus pallidus. The initial low GCS score due to CO exposure appears to be related to the subsequent delayed neurologic sequelae occurrence [9,39,40].

Previous studies have reported that the change in scores is significantly related to the exposure time to CO [30,31]. As the exposure time increases, the volume of oxygen-bound hemoglobin decreases, promoting tissue hypoxia. Therefore, the determination of the average CO exposure time for each study is important in the analysis of the results. However, since many CO exposures occur unintentionally, the patient may be unaware of the precise CO exposure time, and the accuracy of information from patients with CO poisoning who presented with a change in consciousness is limited. In each study included in this meta-analysis, information regarding the CO exposure time was unclear. Additionally, along with the CO exposure time, it is necessary to identify other factors influencing the change in consciousness. In a previous study, when patients with intentional CO poisoning were also compromised by drug use or alcohol intake, it was necessary to differentiate the potential part played by these factors in the change in consciousness [17]. Hampson et al. reported that almost one-half of patients with intentional CO poisoning had ingested one or more poisons in addition to CO [41]. Among them, ethanol was the most common and other sedative-hypnotics were sometimes taken [39]. In another study of accidental and intentional poisonings in Poland, ethanol and CO were combined in 6.2% of cases [42]. Thus, when assessing the mental status of patients with CO poisoning, drug co-ingestion must be considered. It is additionally necessary to investigate the influence of other factors on changes in the GCS scores in patients with CO poisoning.

It is important to predict delayed neurologic sequelae occurrences in advance to prevent it in patients with CO poisoning because a lucid interval precedes the development of delayed neurologic sequelae. Prior studies suggested that hyperbaric oxygen therapy is a possible method for preventing the development of delayed neurologic sequelae in patients with CO poisoning [1,6,13,43,44]. Particularly, Liao et al. reported that a longer duration from CO exposure to hyperbaric oxygen therapy was associated with a higher risk of delayed neurologic sequelae occurrence (odds ratio, 1.06; 95% CI, 1.03–1.09). Additionally, an acceptable timing for hyperbaric oxygen therapy was within 22.5 h after CO poisoning, and hyperbaric oxygen therapy administered >48 h after CO poisoning offered no benefit in terms of prevention of delayed neurologic sequelae [1]. The use of the initial GCS can provide rapid and correct confirmation of patients with a higher risk of delayed neurologic sequelae and inform and promote the immediate implementation of hyperbaric oxygen therapy.

This study had several limitations. First, it may not be appropriate to generalize our results to different healthcare systems and ethnicities, because with the exception of two, all included studies in this meta-analysis were confined to East Asia. For more generally applicable findings, data from other countries and/or ethnicities are required. Second, we limited the abstract searches to studies published in English. Extended studies may require searches for abstracts written in languages other than English in a specific area. Third, we did not perform a detailed analysis of hyperbaric oxygen therapy, which may affect delayed neurologic sequelae occurrence. Because the included studies did not clearly describe the use of hyperbaric oxygen therapy, we were unable to conduct a subgroup analysis of this treatment. Thus, our conclusions are limited because we were unable to identify any detailed methods regarding oxygen therapy and hyperbaric oxygen therapy. Finally, as mentioned earlier, we had insufficient data on CO exposure time and concomitant medications or substances that may have affected the mental status of the patients.

## 5. Conclusions

A low GCS score in patients with early CO poisoning was associated with delayed neurologic sequelae occurrence and could be accessed quickly and easily in the early stage of CO poisoning. In addition, it may be possible to establish an active treatment strategy, such as hyperbaric oxygen therapy, according to GCS scores to minimize neurological sequelae.

## Figures and Tables

**Table 1:** Characteristics of studies included in systematic review and meta-analysis.

Study	Region	Period	Design	Cutoff Point of GCS (Low GCS Score)	No. of Patients (DNS/No DNS)	Age (DNS/No DNS)	Male(n) (DNS/No DNS)	HBOT (DNS/No DNS)	Maximum Time of Assessment of DNS
Liao et al., 2018 [30]	Taiwan	January 2009–December 2015	retrospective, single	<9	62/404	41.0 (26.0–52.0)/ 32.0 (21.0–43.0)	35/195	47/232	6 mon
Huang et al., 2019 [31]	Taiwan	January 2005–December 2014	retrospective, single	<9	93/298	41.4 ± 14.7/ 39.7 ± 14.2	55/163	73/214	6 wks
Liao et al., 2019 [1]	Taiwan	January 2009–December 2015	retrospective, single	<9	48/231	38.4 ± 16.1/33.3 ± 17.1	29/100	48/231	6 mon
Lin et al., 2018 [6]	Taiwan	January 1990–December 2011	retrospective, multicenter	<9	47/591	44.2 ± 12.6/34.3 ± 16.3	27/284	38/505	2 yrs
Pepe et al., 2011 [25]	Italy	1992–2007	retrospective, single	<9	34/107	40.4 ± 15.5/41.7 ± 21.7	11/53	11/44	12 mon
Beppu 2011 [32]	Japan	April 2008–March 2010	prospective, single	<9	3/20	55.0 ± 26.0/54.8 ± 19.2	Not reported	3/20	6 wks
Han et al., 2021 [9]	South Korea	July 2017–February 2020	prospective, single	<10	38/165	44.0 (34.3–57.0)/43.0 (32.0–54.0)	23/113	38/165	6 wks
Lee et al., 2020 [17]	South Korea	January 2018–July 2018	retrospective, single	<10	12/126	47.0 (33.0–50.0)/36.0 (26.0–53.0)	6/69	12/117	6 wks
Beppu 2013 [33]	Japan	April 2008–February 2011	prospective, single	<9	3/19	44.3 ± 13.8/38.4 ± 10.9	Not reported	3/19	6 wks
Gaballah et al., 2020 [19]	Egypt	January 2018–December 2018	prospective, single	<11	10/20	37.0 (18.0–55.0)/23.0 (15.0–40.0)	7/12	2/2	6 mon

Acronyms: GCS, Glasgow coma scale; DNS, delayed neurologic sequelae; and HBOT, hyperbaric oxygenation therapy; wks, weeks; mon, months.

**Table 2:** Subgroup analysis for occurrence of delayed neurologic sequelae.

Characteristics	Development of DNS			
	*n*	OR (95%CI)	*p*-Value for Heterogeneity	I^2^ (%)
All studies				
All	10	2.98 (2.10–4.23)	0.14	33
Sample size				
≥100	7	2.79 (1.98–3.93)	0.16	36
<100	3	8.31 (2.07–33.43)	0.43	0
Cutoff point (low GCS score group)				
<9	7	2.80 (1.91–4.12)	0.17	34
<10 or <11	3	4.24 (1.55–11.56)	0.15	48
Study quality				
High	6	2.66 (1.83–3.88)	0.14	40
Low	4	4.86 (2.36–10.00)	0.48	0

Acronyms: GCS, Glasgow coma scale; DNS, delayed neurologic sequelae; and OR, Odd ratios.

## Data Availability

The datasets generated during the current study are available from the corresponding author on reasonable request.

## Supplementary Materials

The following supporting information can be downloaded at: https://www.mdpi.com/article/10.3390/jpm12040635/s1, Table S1: Search strategy; Table S2: List of excluded references after full-text review; Table S3: Egger’s regression test for publication bias; Figure S1: Risk of bias and quality assessment of included studies by the QUIPS tool; Figure S2: Funnel plot for publication bias.
