# Supplementary material for: Association between Glasgow Coma Scale in Early Carbon Monoxide Poisoning and Development of Delayed Neurological Sequelae: A Meta-Analysis
Source: J Pers Med. 2022 Apr 14;12(4):635. doi: 10.3390/jpm12040635 (PMC9031955; doi:10.3390/jpm12040635)
Supplement: Supplementary file 1 [file jpm-12-00635-s001.zip › jpm-1580297-supplementary.pdf]

Journal of
Personalized Medicine

MDPI

Supplementary Materials

Table S1. Search strategy.

Medline
#1. exp Carbon Monoxide OR exp Carbon Monoxide Poisoning OR carbon monoxide poisoning.ti,ab,kw OR carbon monoxide intoxication.ti,ab,kw OR CO poisoning.ti,ab,kw OR CO intoxication.ti,ab,kw<br>#2. exp Cognition/ or exp Nervous System Diseases/ or exp Cognition Disorders/ OR exp Cognitive Dysfunction/ OR delayed neuropsychological sequelae.ti,ab,kw OR delayed neurological sequelae.ti,ab,kw OR DNS.ti,ab,kw<br>#1 AND #2
Embase
#1. 'carbon monoxide'/exp OR 'carbon monoxide intoxication'/exp OR 'carbon monoxide':ab,kw,ti OR 'carbon monoxide intoxication':ab,kw,ti OR 'carbon monoxide poisoning':ab,kw,ti OR 'co poisoning':ab,kw,ti OR 'co intoxication':ab,kw,ti<br>#2. 'neurologic disease'/exp OR 'neuropsychology'/exp OR 'cognition'/exp OR 'cognitive defect'/exp OR 'delayed neurotoxicity'/exp OR 'neurologic disease'/exp OR 'neuropsychology'/exp OR 'cognition'/exp OR 'cognitive defect'/exp OR 'delayed neurotoxicity'/exp OR 'delayed neurological sequelae':ti,ab,kw OR 'dns':ti,ab,kw<br>#1 AND #2
Cochrane library
#1. Carbon Monoxide [MeSH] OR Carbon Monoxide [MeSH] OR carbon monoxide poisoning OR carbon monoxide intoxication<br>#2. Congnition [MeSH] OR Nervous System Diseases [MeSH] OR Cognition Disorders [MeSH] OR delayed neurological sequelae OR delayed neuropsychological sequelae #1 AND #2

Table S2. List of excluded references after full-text review.

Title	First Author	Journal (Year)	Main Reason for Exclusion
Longitudinal gray matter changes of the pain matrix in patients with carbon monoxide intoxication: A voxel-based morphometry study	Chou et al.	European Journal of Radiology (2020)	Intervention was not intended for initial GCS
S-100beta in predicting the need of hyperbaric oxygen in CO-induced delayed neurological sequels	Hafez et al. [14]	Human & Experimental Toxicology (2020)	Intervention was not intended for initial GCS
Predictive factors for acute brain lesions on magnetic resonance imaging in acute carbon monoxide poisoning	Kim et al.	American Journal of Emergency Medicine (2020)	Intervention was not intended for initial GCS
Carbon monoxide poisoning-induced delayed encephalopathy accompanies decreased microglial cell numbers: Distinctive pathophysiological features from hypoxemia induced brain damage	Sekiya et al. [4]	Brain Research (2019)	Animal study
Evaluation of changes in magnetic resonance diffusion tensor imaging after treatment of delayed encephalopathy due to carbon monoxide poisoning	Wu et al.	Journal of Integrative Neuroscience (2019)	Intervention was not intended for initial GCS
Gray matter nuclei damage in acute carbon monoxide intoxication assessed in vivo using diffusion tensor MR imaging	Jiang et al.	Radiologia Medica (2020)	Intervention was not intended for initial GCS
Serum lactate as a predictor of neurologic outcome in ED patients with acute carbon monoxide poisoning	Jung et al.	American Journal of Emergency Medicine (2019)	Outcome also included persistent neurological sequelae (PNS)
Cerebral Damage after Carbon Monoxide Poisoning: A Longitudinal Diffusional Kurtosis Imaging Study	Zhang et al. [7]	American Journal of Neuroradiology (2019)	Intervention was not intended for initial GCS
Acute Brain Lesions on Magnetic Resonance Imaging and Delayed Neurological Sequelae in Carbon Monoxide Poisoning	Jeon et al. [21]	JAMA Neurology (2018)	Intervention was not intended for initial GCS
The impact of hyperthermia after acute carbon monoxide poisoning on neurological sequelae	Moon et al.	Human & Experimental Toxicology (2019)	Outcome also included persistent neurological sequelae (PNS)
Clinical Predictors of Acute Brain Injury in Carbon Monoxide Poisoning Patients with Altered Mental Status at Admission to Emergency Department	Kim et al.	Academic Emergency Medicine (2019)	Intervention was not intended for initial GCS
Neuroprotective effect of ethanol in acute carbon monoxide intoxication: A retrospective study	Kim et al.	Medicine (2018)	Intervention was not intended for initial GCS
Serum neuron-specific enolase levels at presentation and long-term neurological sequelae after acute charcoal burning-induced carbon monoxide poisoning	Moon et al.	Clinical Toxicology (2018)	Intervention was not intended for initial GCS
Neurotoxicity of carbon monoxide targets caudate mediated dopaminergic system	Sun et al.	Neurotoxicology (2018)	Intervention was not intended for initial GCS
Serum N-terminal proBNP, not troponin I, at presentation predicts long-term neurologic outcome in acute charcoal-burning carbon monoxide intoxication	Moon et al.	Clinical Toxicology (2018)	DNS was not described
The usefulness of diffusion weighted magnetic resonance imaging performed in the acute phase as an early predictor of delayed neuropsychiatric sequelae in acute carbon monoxide poisoning	Kim et al. [22]	Human & Experimental Toxicology (2018)	Intervention was not intended for initial GCS
Initial diffusion-weighted MRI and long-term neurologic outcomes in charcoal-burning carbon monoxide poisoning	Moon et al.	Clinical Toxicology (2018)	Intervention was not intended for initial GCS
Serum neuron-specific enolase as an early predictor of delayed neuropsychiatric sequelae in patients with acute carbon monoxide poisoning	Cha et al. [20]	Human & Experimental Toxicology (2018)	Intervention was not intended for initial GCS
Efficacy of N-Butylphthalide and Hyperbaric Oxygen Therapy on Cognitive Dysfunction in Patients with Delayed Encephalopathy After Acute Carbon Monoxide Poisoning	Xiang et al.	Medical Science Monitor (2017)	Intervention was not intended for initial GCS

Combined application of dexamethasone and hyperbaric oxygen therapy yields better efficacy for patients with delayed encephalopathy after acute carbon monoxide poisoning	Xiang et al.	Drug design, development & therapy (2017)	Control groups were not eligible for comparison with intervention groups
MRI and clinical manifestations of delayed encephalopathy after carbon monoxide poisoning	Wang et al.	Pakistan Journal of Pharmaceutical Sciences (2016)	Control groups were not eligible for comparison with intervention groups
Reversible Changes of Brain Perfusion SPECT for Carbon Monoxide Poisoning-Induced Severe Akinetic Mutism	Chen et al.	Clinical Nuclear Medicine (2016)	Control groups were not eligible for comparison with intervention groups
Increased Long-Term Risk of Dementia in Patients with Carbon Monoxide Poisoning: A Population-Based Study	Wong et al.	Medicine (2016)	DNS was not described.
Acute carbon monoxide poisoning in a regional hospital in Hong Kong: historical cohort study	Chan et al.	Hong Kong Medical Journal (2016)	Intervention was not intended for initial GCS
Altered white matter metabolism in delayed neurologic sequelae after carbon monoxide poisoning: A proton magnetic resonance spectroscopic study	Kuroda et al.	Journal of the Neurological Sciences (2016)	Intervention was not intended for initial GCS
Delayed visual disturbances in carbon monoxide poisoning: Identification and evaluation	Stabile et al.	Undersea & Hyperbaric Medicine (2015)	Intervention was not intended for initial GCS
Increased risk of Parkinson disease in patients with carbon monoxide intoxication: a population-based cohort study	Lai et al.	Medicine (2015)	Intervention was not intended for initial GCS
Cognitive severity-specific neuronal degenerative network in charcoal burning suicide-related carbon monoxide intoxication: a multimodality neuroimaging study in Taiwan	Chen et al.	Medicine (2015)	Intervention was not intended for initial GCS
Novel clinical grading of delayed neurologic sequelae after carbon monoxide poisoning and factors associated with outcome	Kuroda et al.	Neurotoxicology (2015)	Intervention was not intended for initial GCS
Lack of pupil reflex and loss of consciousness predict 30-day neurological sequelae in patients with carbon monoxide poisoning	Zou et al. [34]	PLoS ONE (2015)	Intervention was not intended for initial GCS
18F-FP-(+)-DTBZ positron emission tomography detection of monoaminergic deficient network in patients with carbon monoxide related parkinsonism	Chang et al.	European Journal of Neurology (2015)	Intervention was not intended for initial GCS
Neuropsychological performance in patients with carbon monoxide poisoning	Yeh et al.	Applied Neuropsychology: Adult (2014)	Intervention was not intended for initial GCS
Plasma copeptin as a predictor of intoxication severity and delayed neurological sequelae in acute carbon monoxide poisoning	Pang et al. [35]	Peptides (2014)	Intervention was not intended for initial GCS
A positive Babinski reflex predicts delayed neuropsychiatric sequelae in Chinese patients with carbon monoxide poisoning	Zou et al.	BioMed Research International (2014)	Intervention was not intended for initial GCS
The impacts of acute carbon monoxide poisoning on the brain: Longitudinal clinical and 99mTc ethyl cysteinate brain SPECT characterization of patients with persistent and delayed neurological sequelae	Tsai et al.	Clinical Neurology & Neurosurgery (2014)	Intervention was not intended for initial GCS
The role of S100B protein, neuron-specific enolase, and glial fibrillary acidic protein in the evaluation of hypoxic brain injury in acute carbon monoxide poisoning	Akdemir et al.	Human & Experimental Toxicology (2014)	DNS was not described
Predictors for delayed encephalopathy following acute carbon monoxide poisoning	Kudo et al. [16]	BMC Emergency Medicine (2014)	Intervention was not intended for initial GCS
A PARK2 polymorphism associated with delayed neuropsychological sequelae after carbon monoxide poisoning	Liang et al.	BMC Medical Genetics (2013)	Intervention was not intended for initial GCS
Neuroprotective effects of erythropoietin in patients with carbon monoxide poisoning	Pang et al.	Journal of Biochemical & Molecular Toxicology (2013)	Intervention was not intended for initial GCS

Diffusion tensor imaging for predicting the clinical outcome of delayed encephalopathy of acute carbon monoxide poisoning	Hou et al.	European Neurology (2013)	Intervention was not intended for initial GCS
Detection of gray matter damage using brain MRI and SPECT in carbon monoxide intoxication: a comparison study with neuropsychological correlation	Chen et al.	Clinical Nuclear Medicine (2013)	Intervention was not intended for initial GCS
Diffusion-weighted imaging improves prediction in cognitive outcome and clinical phases in patients with carbon monoxide intoxication	Chen et al.	Neuroradiology (2013)	Intervention was not intended for initial GCS
Delayed parkinsonism after CO intoxication: evaluation of the substantia nigra with inversion recovery MR imaging	Kao et al.	Radiology (2012)	Intervention was not intended for initial GCS
The usefulness of the serum s100b protein for predicting delayed neurological sequelae in acute carbon monoxide poisoning	Park et al. [15]	Clinical Toxicology (2012)	Intervention was not intended for initial GCS
Striatal dopamine transporter binding for predicting the development of delayed neuropsychological sequelae in suicide attempters by carbon monoxide poisoning: A SPECT study	Yang et al.	Psychiatry Research (2011)	Intervention was not intended for initial GCS
Neuropsychiatric disorders and risk factors in carbon monoxide intoxication	Katirci et al.	Toxicology & Industrial Health (2011)	DNS was not described
Factors affecting the prognosis of patients with delayed encephalopathy after acute carbon monoxide poisoning	Hu et al.	American Journal of Emergency Medicine (2011)	Intervention was not intended for initial GCS
Predictors of carbon monoxide poisoning-induced delayed neuropsychological sequelae	Ku et al.	General Hospital Psychiatry (2010)	Intervention was not intended for initial GCS
Hyperbaric oxygen ameliorates delayed neuropsychiatric syndrome of carbon monoxide poisoning	Chang et al.	Undersea & Hyperbaric Medicine (2010)	Intervention was not intended for initial GCS
Longitudinal study of carbon monoxide intoxication by diffusion tensor imaging with neuropsychiatric correlation	Chang et al.	Journal of Psychiatry & Neuroscience (2010)	Intervention was not intended for initial GCS
Assessment of damage to cerebral white matter fiber in the subacute phase after carbon monoxide poisoning using fractional anisotropy in diffusion tensor imaging	Beppu et al.	Neuroradiology (2010)	Intervention was not intended for initial GCS
S-100beta and neuron-specific enolase levels in carbon monoxide-related brain injury	Cakir et al.	American Journal of Emergency Medicine (2010)	DNS was not described
The early elevation of interleukin 6 concentration in cerebrospinal fluid and delayed encephalopathy of carbon monoxide poisoning	Ide et al.	American Journal of Emergency Medicine (2009)	Control groups were not eligible
Elevated serum S100B protein and neuron-specific enolase levels in carbon monoxide poisoning	Yardan et al.	American Journal of Emergency Medicine (2009)	DNS was not described
Damage of white matter tract correlated with neuropsychological deficits in carbon monoxide intoxication after hyperbaric oxygen therapy	Chang et al.	Journal of Neurotrauma (2009)	Intervention was not intended for initial GCS
Myelin basic protein in cerebrospinal fluid: a predictive marker of delayed encephalopathy from carbon monoxide poisoning	Ide et al.	American Journal of Emergency Medicine (2008)	Control groups were not eligible
Cognitive and affective outcomes of more severe compared to less severe carbon monoxide poisoning	Chambers et al. [8]	Brain Injury (2008)	Intervention was not intended for initial GCS
Carbon monoxide poisoning: risk factors for cognitive sequelae and the role of hyperbaric oxygen	Weaver et al. [5]	American Journal of Respiratory & Critical Care Medicine (2007)	Intervention was not intended for initial GCS
Delayed encephalopathy after carbon monoxide intoxication-long-term prognosis and correlation of clinical manifestations and neuroimages	Hsiao et al.	Acta Neurologica Taiwanica (2004)	Intervention was not intended for initial GCS
S100B protein in carbon monoxide poisoning: a pilot study	Brvar et al.	Resuscitation (2004)	Intervention was not intended for initial GCS
Diffusion-weighted MRI and 99mTc-HMPAO SPECT in delayed relapsing type of carbon monoxide poisoning: evidence of delayed cytotoxic edema	Chu et al.	European Neurology (2004)	Intervention was not intended for initial GCS

Memory one month after acute carbon monoxide intoxication: a prospective study	Deschamps et al.	Occupational & Environmental Medicine (2003)	Intervention was not intended for initial GCS
Parkinsonism after carbon monoxide poisoning	Choi et al.	European Neurology (2002)	Intervention was not intended for initial GCS
White matter hyperintensities and neuropsychological outcome following carbon monoxide poisoning	Parkinson et al.	Neurology (2002)	Intervention was not intended for initial GCS
Verbal memory deficits associated with fornix atrophy in carbon monoxide poisoning	Kesler et al.	Journal of the International Neuropsychological Society (2001)	Intervention was not intended for initial GCS
Severe carbon monoxide poisoning: outcome after hyperbaric oxygen therapy	Hawkins et al.	British Journal of Anaesthesia (2000)	DNS was not described.
The magnetic resonance imaging appearances of the brain in acute carbon monoxide poisoning	O'Donnell et al.	Clinical Radiology (2000)	DNS was not described.
Clinical outcome and magnetic resonance imaging of carbon monoxide intoxication. A long-term follow-up study	Pavese et al.	Italian Journal of Neurological Sciences (1999)	Intervention was not intended for initial GCS
Delayed movement disorders after carbon monoxide poisoning	Choi et al.	European Neurology (1999)	Intervention was not intended for initial GCS
Regional cerebral blood flow measurements with Xenon-CT in the prediction of delayed encephalopathy after carbon monoxide intoxication	Sesay et al.	Acta Neurologica Scandinavica (1996)	Intervention was not intended for initial GCS
Delayed neuropsychologic sequelae after carbon monoxide poisoning: prevention by treatment with hyperbaric oxygen	Thom et al.	Annals of Emergency Medicine (1995)	Intervention was not intended for initial GCS
Evaluation of outcome of delayed neurologic sequelae after carbon monoxide poisoning by technetium-99m hexamethylpropylene amine oxime brain single photon emission computed tomography	Choi et al.	European Neurology (1995)	Intervention was not intended for initial GCS
Computed tomographic findings after acute carbon monoxide poisoning	Jones et al.	American Journal of Emergency Medicine (1994)	DNS was not described.
Neurological sequelae following carbon monoxide poisoning clinical course and outcome according to the clinical types and brain computed tomography scan findings	Lee et al.	Movement Disorders (1994)	Intervention was not intended for initial GCS
Technetium-99m HM-PAO SPECT in patients with delayed neurologic sequelae after carbon monoxide poisoning	Choi et al.	Journal of Korean Medical Science (1992)	Intervention was not intended for initial GCS
A brain syndrome associated with delayed neuropsychiatric sequelae following acute carbon monoxide intoxication	Min et al.	Acta Psychiatrica Scandinavica (1986)	Intervention was not intended for initial GCS
Delayed neurologic sequelae in carbon monoxide intoxication	Choi . [11]	Archives of Neurology (1983)	Intervention was not intended for initial GCS
Mechanism of delayed encephalopathy after acute carbon monoxide poisoning	Huang et al.	Neural Regeneration Research (2020)	Animal study
Glasgow Coma Scale is a better delayed neurological sequelae risk factor than neurological examination abnormalities in carbon monoxide poisoning	Xu et al.	American Journal of Emergency Medicine (2020)	Intervention was not intended for initial GCS
Cerebral White Matter Lesions on Diffusion-Weighted Images and Delayed Neurological Sequelae after Carbon Monoxide Poisoning: A Prospective Observational Study	Nah et al. [23]	Diagnostics (2020)	Intervention was not intended for initial GCS
Selective Susceptibility of Oligodendrocytes to Carbon Monoxide Poisoning: Implication for Delayed Neurologic Sequelae (DNS)	Tian et al.	Frontiers in Psychiatry (2020)	Control groups were not eligible
Study on brain structure network of patients with delayed encephalopathy after carbon monoxide poisoning: based on diffusion tensor imaging	Jiang et al.	Nuroradiology (2020)	Intervention was not intended for initial GCS
Abnormal degree centrality in delayed encephalopathy after carbon monoxide poisoning: a resting-state fMRI study	Wu et al.	Neuroradiology (2020)	Intervention was not intended for initial GCS
The Cannabinoid WIN 55,212-2 Reduces Delayed Neurologic Sequelae After Carbon Monoxide Poisoning by Promoting Microglial M2 Polarization Through ST2 Signaling	Du et al.	Journal of Molecular Neuroscience (2020)	Animal study

Oligodendrocyte dysfunction and regeneration failure: A novel hypothesis of delayed encephalopathy after carbon monoxide poisoning	Guo et al.	Medical Hypotheses (2020)	Control groups were not eligible
The efficacy of N-butylphthalide and dexamethasone combined with hyperbaric oxygen on delayed encephalopathy after acute carbon monoxide poisoning	Zhang et al.	Drug Design, Development and Therapy (2020)	Intervention was not intended for initial GCS
Serum netrin-1 levels at presentation and delayed neurological sequelae in unintentional carbon monoxide poisoning	Kokulu et al.	Clinical Toxicology (2020)	Intervention was not intended for initial GCS
Assessment of serum glucose/potassium ratio as a predictor for delayed neuropsychiatric syndrome of carbon monoxide poisoning	Demirtaş et al.	Human and Experimental Toxicology (2020)	Intervention was not intended for initial GCS
Multicenter retrospective analysis of the risk factors for delayed neurological sequelae after acute carbon monoxide poisoning	Zhang et al.	American Journal of Emergency Medicine (2020)	Intervention was not intended for initial GCS
Association between Neuron-Specific Enolase Gene Polymorphism and Delayed Encephalopathy after Acute Carbon Monoxide Poisoning	Xu et al.	Behavioural Neurology (2020)	Intervention was not intended for initial GCS
Hyperbaric Oxygen Therapy Did Not Prevent Delayed Neuropsychiatric Sequelae in a Prospective Observational Study with Propensity Score Matching in 224 Patients With Acute Carbon Monoxide Toxicity	Han et al.	Journal of Emergency Medicine (2020)	Intervention was not intended for initial GCS
LRCH1 polymorphisms linked to delayed encephalopathy after acute carbon monoxide poisoning identified by GWAS analysis followed by Sequenom MassARRAY® validation	Gu et al.	BMC Medical Genetics (2019)	Intervention was not intended for initial GCS
Utility of brain CT for predicting delayed encephalopathy after acute carbon monoxide poisoning	Du et al. [39]	Experimental and Therapeutic Medicine (2019)	Intervention was not intended for initial GCS
Longitudinal white matter changes following carbon monoxide poisoning: A 9-month follow-up voxelwise diffusional kurtosis imaging study	Chou et al.	American Journal of Neuroradiology (2019)	Intervention was not intended for initial GCS
Demographic characteristics and delayed neurological sequelae risk factors in carbon monoxide poisoning	Sarı Doğan et al.	American Journal of Emergency Medicine (2019)	Intervention was not intended for initial GCS
Impact of hyperbaric oxygen therapy on subsequent neurological sequelae following carbon monoxide poisoning	Huang et al.	Journal of Clinical Medicine (2018)	Intervention was not intended for initial GCS
Diffusion kurtosis imaging as a neuroimaging biomarker in patients with carbon monoxide intoxication	Lee et al.	NeuroToxicology (2018)	Intervention was not intended for initial GCS
Dynamic changes and clinical significance of serum S100B protein and glial fibrillary acidic protein in patients with delayed encephalopathy after acute carbon monoxide poisoning	Di et al.	Pakistan Journal of Medical Sciences (2018)	Intervention was not intended for initial GCS
Efficacy of combined glucocorticoid and hyperbaric oxygen therapy against delayed encephalopathy after carbon monoxide poisoning, and its effect on expression of immune-associated cytokines	Li et al.	Tropical Journal of Pharmaceutical Research (2018)	Control groups were not eligible for comparison with intervention groups
Clinical features and risk factors analysis of delayed encephalopathy after acute carbon monoxide poisoning	Tianhong et al.	Acta Medica Mediterranea (2018)	Intervention was not intended for initial GCS
Variability in Treatment for Carbon Monoxide Poisoning in Japan: A Multicenter Retrospective Survey	Fujita et al.	Emergency Medicine International (2018)	DNS was not described
Copeptin levels in carbon monoxide poisoning	İrem et al.	Turkish Journal of Medical Sciences (2017)	DNS was not described
Efficacy of Combined XingZhi-YiNao Granules and Hyperbaric Oxygen Therapy for Cognition and Motor Dysfunction in Patients with Delayed Encephalopathy after Acute Carbon Monoxide Poisoning	Qin et al.	Evidence-based Complementary and Alternative Medicine (2017)	Intervention was not intended for initial GCS
Risk factors for the delayed onset of neuropsychologic sequelae following carbon monoxide poisoning	Kitamoto et al. [18]	Acute Medicine and Surgery (2016)	Intervention was not intended for initial GCS Creatinine kinase

Lp-PLA2 variants associated with delayed encephalopathy after acute carbon monoxide poisoning	Zhao et al.	International Journal of Clinical and Experimental Medicine (2016)	Intervention was not intended for initial GCS
Metabolic Covariant Network in Relation to Nigrostriatal Degeneration in Carbon Monoxide Intoxication-Related Parkinsonism	Chang et al.	Frontiers in Neuroscience (2016)	Intervention was not intended for initial GCS
Ischemia-modified albumin levels in the prediction of acute critical neurological findings in carbon monoxide poisoning	Daş et al.	Kaohsiung Journal of Medical Sciences (2016)	DNS was not described
Can initial lactate levels predict the severity of unintentional carbon monoxide poisoning?	Doɨan et al.	Human and Experimental Toxicology (2015)	Intervention was not intended for initial GCS
Predicting poor outcome in patients with intentional carbon monoxide poisoning and acute respiratory failure: A retrospective study	Shen et al.	Journal of Medical Sciences (Taiwan) (2015)	Outcome also included persistent neurological sequelae (PNS)
Increased risk of Parkinson disease in patients with carbon monoxide intoxication	Lai et al.	Medicine (United States) (2015)	Control groups were not eligible for comparison with intervention groups
Neuropsychological outcome after carbon monoxide exposure following a storm: A case-control study	Pages et al.	BMC Neurology (2014)	Control groups were not eligible for comparison with intervention groups
Detecting damaged regions of cerebral white matter in the subacute phase after carbon monoxide poisoning using voxel-based analysis with diffusion tensor imaging	Fujiwara et al.	Neuroradiology (2012)	Outcome also included persistent neurological sequelae (PNS)
Elevated S100B level in cerebrospinal fluid could predict poor outcome of carbon monoxide poisoning	Ide et al.	American Journal of Emergency Medicine (2012)	Intervention was not intended for initial GCS
The value of initial lactate in patients with carbon monoxide intoxication: In the emergency department	Moon et al.	Human and Experimental Toxicology (2011)	Intervention was not intended for initial GCS
Hydrogen-rich saline reduces delayed neurologic sequelae in experimental carbon monoxide toxicity	Sun et al.	Critical Care Medicine (2011)	Animal study
Tc99m-sestamibi thigh SPECT/CT images for noninvasive assessment of skeletal muscle injury in carbon monoxide intoxication with clinical and pathological correlation	Huang et al.	Clinical Nuclear Medicine (2011)	Control groups were not eligible for comparison with intervention groups
Clinical significance of the pallidoreticular pathway in patients with carbon monoxide intoxication	Chang et al.	Brain (2011)	Control groups were not eligible for comparison with intervention groups
White matter damage in carbon monoxide intoxication assessed in vivo using diffusion tensor MR imaging	Lin et al.	American Journal of Neuroradiology (2009)	Control groups were not eligible for comparison with intervention groups.
Comparison of SPECT findings and neuropsychological sequelae in carbon monoxide and organophosphate poisoning	Ozyurt et al.	Clinical Toxicology (2008)	Control groups were not eligible for comparison with intervention groups
Affective outcome following carbon monoxide poisoning: A prospective longitudinal study	Jasper et al.	Cognitive and Behavioral Neurology (2005)	DNS was not described.
Is elevated plasma lactate a useful marker in the evaluation of pure carbon monoxide poisoning?	Benaissa et al.	Intensive Care Medicine (2003)	Intervention was not intended for initial GCS
Quantitative PET scan findings in carbon monoxide poisoning: Deficits seen in a matched pair	Pinkston et al.	Archives of Clinical Neuropsychology (2000)	Intervention was not intended for initial GCS
A long-term follow-up study of serial magnetic resonance images in patients with delayed encephalopathy after acute carbon monoxide poisoning	Inagaki et al.	Psychiatry and Clinical Neurosciences (1997)	Intervention was not intended for initial GCS comparison with intervention groups

Computed tomography of the brain in acute carbon monoxide poisoning	Silver et al.	Clinical Radiology (1996)	Intervention was not intended for initial GCS
A longitudinal study of 100 consecutive admissions for carbon monoxide poisoning to the Royal Adelaide Hospital	Gorman et al.	Anaesthesia and Intensive Care (1992)	DNS was not described
Hematological parameters as early predictors of delayed neurological sequelae in acute carbon monoxide poisoning	Shahin et al.	Ain Shams Journal of Forensic Medicine and Clinical Toxicology (2020)	Intervention was not intended for initial GCS

Acronyms: CO, carbon monoxide; GCS, Glasgow coma scale; DNS, delayed neurologic sequelae.

Table S3. Egger's regression test for publication bias.

Statistic	Intercept	Confidence Interval	T	*p*-value
Egger's test	1.1558	(−0.4190)–2.7306	1.44	0.1883

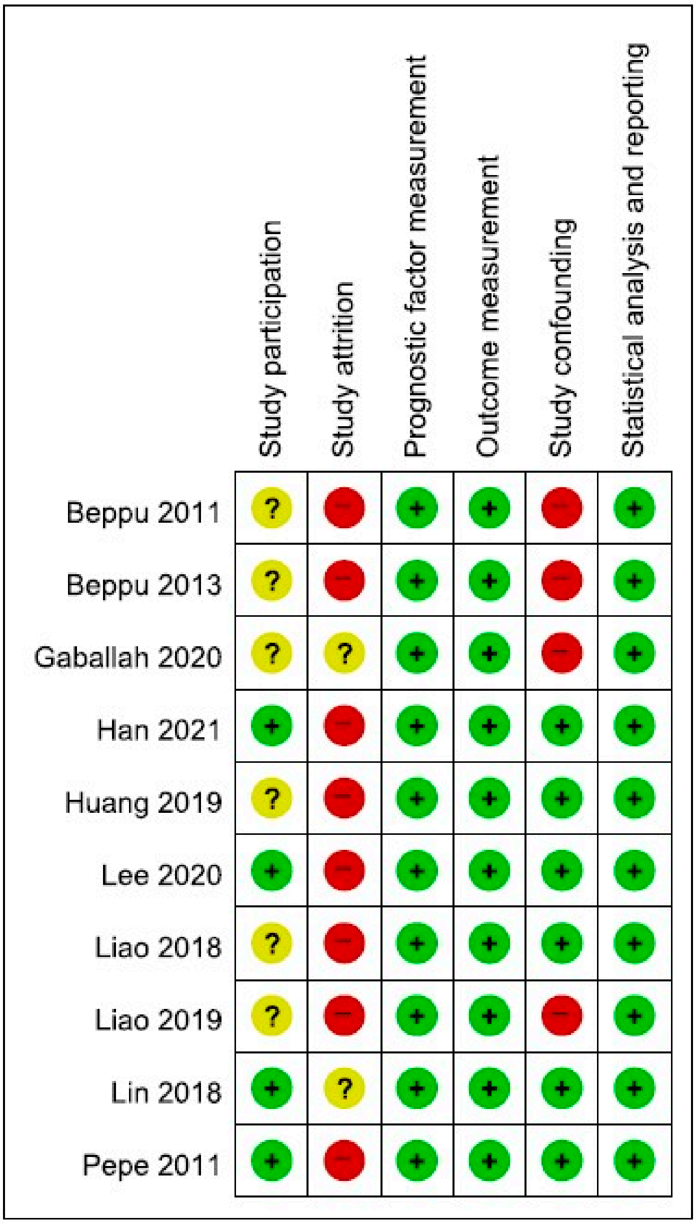

Figure S1. Risk of bias and quality assessment of included studies by the QUIPS tool. +, low; -, high; ?, unclear. [1,6,9,17,19,25,30–33].

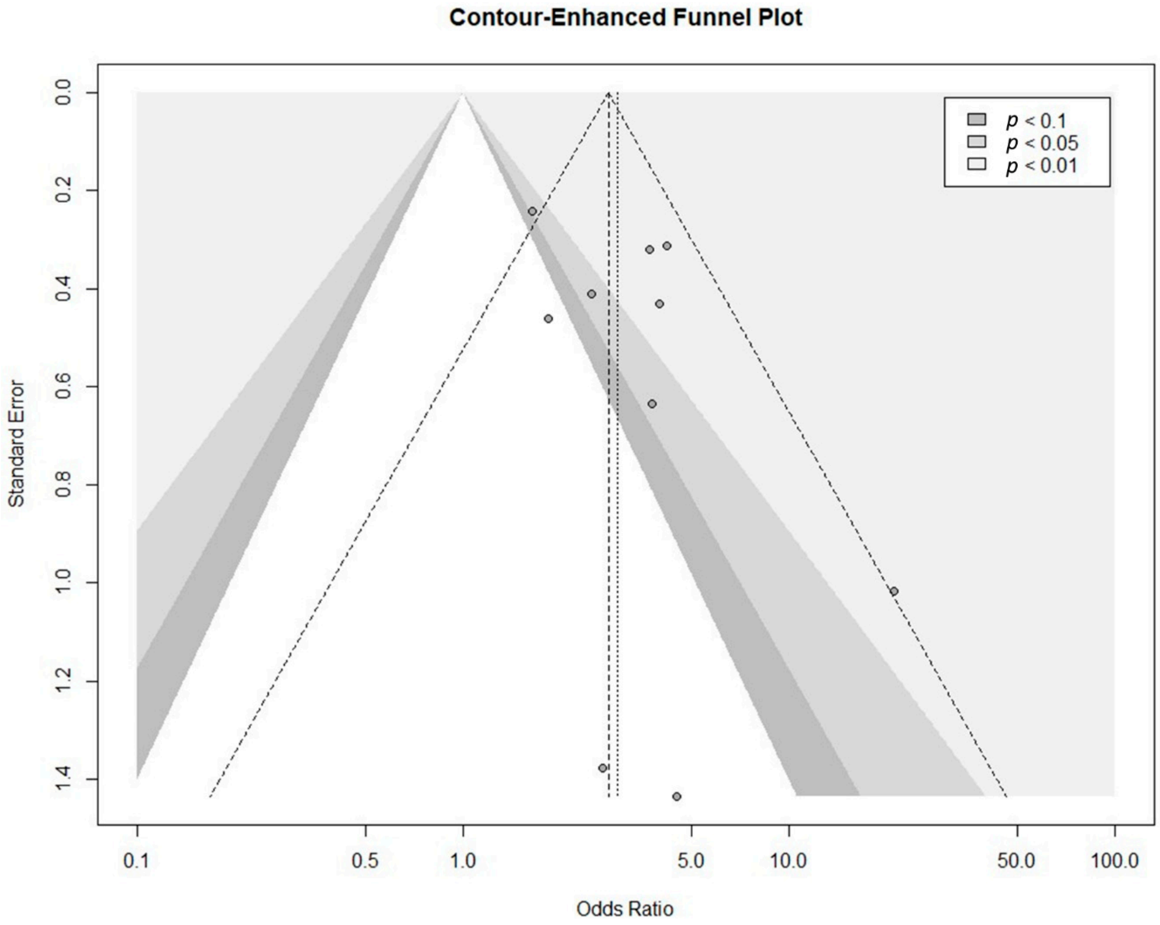

Figure S2. Funnel plot for publication bias.
